# Kanamycin detection at graphene quantum dot-decorated gold nanoparticles in organized medium after solid-phase extraction using an aminoglycoside imprinted polymer

**DOI:** 10.1016/j.mex.2018.11.019

**Published:** 2018-11-30

**Authors:** Carlos A.T. Toloza, Joseany M.S. Almeida, Sarzamin Khan, Yasmin G. dos Santos, Andrea R. da Silva, Ricardo Q. Aucélio

**Affiliations:** a Department of Chemistry, Pontifical Catholic University of Rio de Janeiro (PUC-Rio), Rio de Janeiro, 22451-900, Brazil; b Chemistry Program, Universidad del Atlantico, Puerto Colombia, Colombia; c Department of Chemistry, University of Swabi, Khyber Pakhtunkhwa, Anbar, 23561, Pakistan; d Centro Federal de Educação Tecnológica Celso Suckow da Fonseca (CEFET/RJ), 27600-000, Valença, RJ, Brazil

**Keywords:** Photoluminescence spectrometry, Solid-phase extraction, Nanoparticle based analytical probing, Amino-functionalized graphene quantum dots, Gold nanoparticles, Imprinted polymer, Photoluminescent nanoprobe, Kanamycin sulfate

## Abstract

This is a description of the indirect determination of kanamycin sulfate though the photoluminescence enhancement of an aqueous dispersion of amino-functionalized graphene quantum dots (amino-GQDs) coupled with gold nanoparticles (AuNPs) in a cationic surfactant-rich medium. Specifically, cetyltrimethylammonium bromide (CTAB) was used as the cationic surfactant in our work. Previously, solid phase extraction with a cartridge packed with aminoglycoside-selective imprinted polymer ensured selectivity in kanamycin determination in yellow-fever vaccine and veterinary pharmaceutical samples. The proposed method has trace analysis capability and it is simple to perform as it does not involve the use of toxic reagents employed for chemical derivatization of aminoglycoside antibiotics.

## Graphical abstract

**Figure:**
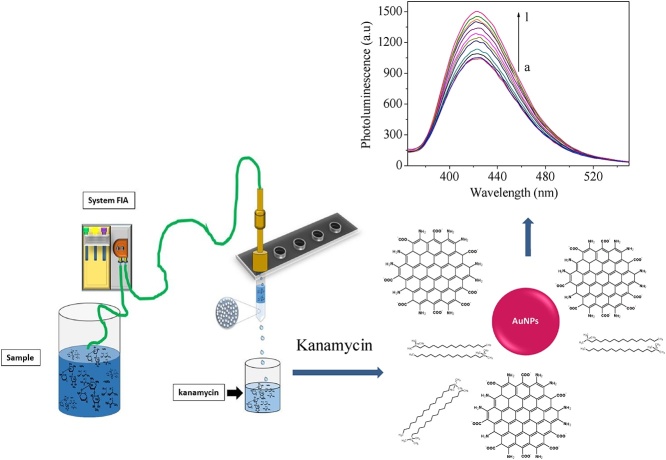

**Specifications Table**

**Table:** 

*Subject area*	Analytical Chemistry
*Method names*	Photoluminescence spectrometry, solid-phase extraction, nanoparticle based analytical probing

## Method details

Previous work has shown the importance of the indirect determination of kanamycin sulfate using semiconductor quantum dots and gold nanoparticles [1]. In this paper, a recently published experimental procedure, used to the selective determination of kanamycin sulfate [2], is reported. The indirect determination was made by measuring the photoluminescence response of an off-on probe based on spherical gold nanoparticles (AuNPs) decorated with amino-functionalized graphene quantum dots (amino-GQDs) obtained from the exfoliation of a mixture of citric acid and glutathione (GSH) in an aqueous solution containing hexadecyltrimethylammonium bromide (CTAB). The CTAB is used as a bridge to facilitate the approximation of the negatively charged AuNPs on the surface and the amino-GQDs, which are also negatively charged. The presence of CTAB in the AuNPs-amino-GQDs system also helped to improve the interaction of kanamycin with the nanostructure [2]. In the presence of kanamycin sulfate, the signal of a photoluminescence suppressed nanoparticle system (AuNPs-amino-GQDs-CTAB) was restored. Characterization of the AuNP-amino-GQD-CTAB system [2] and the optimization of its synthesis are reported in this paper. Solid-phase extraction using a laboratory-made kanamycin sulfate imprinted polymer was used to facilitate previous separation of analyte from samples and offer selectivity by the method.

## Reagents

Ultrapure water (18.2 MΩ cm) was from the Milli-Q gradient A10 ultra-purifier (Milipore, USA). Reduced l-glutathione (GSH), kanamycin sulfate, hydrogen tetrachloroaurate(III) hydrate, (3-aminopropyl) trimethoxysilane (APTMS), tetraethyl orthosilicate (TEOS), CTAB, were from Sigma-Aldrich (USA). Sodium hydroxide, hydrochloric acid and citric acid were from Merck (Germany). Sodium borohydride (NaBH_4_) was from Fluka.

## Materials

Syringe filters (0.22 μm) were from Whatman, UK. Dialysis membrane (retained molecular weight of 3.5 kDa) was from Spectrum Laboratories Inc. (USA).

## Apparatuses

### Photoluminescence

Photoluminescence measurements were made on a model LS 55 luminescence spectrometer (Perkin-Elmer) using a 10.0 nm spectral bandpass and 1 cm optical path length quartz cuvettes. A thermostatic system with stirring (PTP-1 Fluorescence Peltier System with a PCB1500Water Peltier System, Perkin-Elmer) was used to keep the dispersions in the cuvette at specific temperatures during photoluminescence measurements.

### Solid-phase extraction (SPE)

A FIAlab-2500 (FIAlab Instrument, USA) flow injection system was used to feed the SPE cartridge at a constant rate of solvents during conditioning, cleaning and elution steps.

### Synthesis of nanoparticle system

An Orb jacketed reactor system (model R18, Syrris Ltd, UK) was used to produce an aqueous dispersion of AuNP-amino-GQDs-CTAB.

## Procedures

### Synthesis of aqueous dispersion of amino-GQDs

**Fig. 1:**
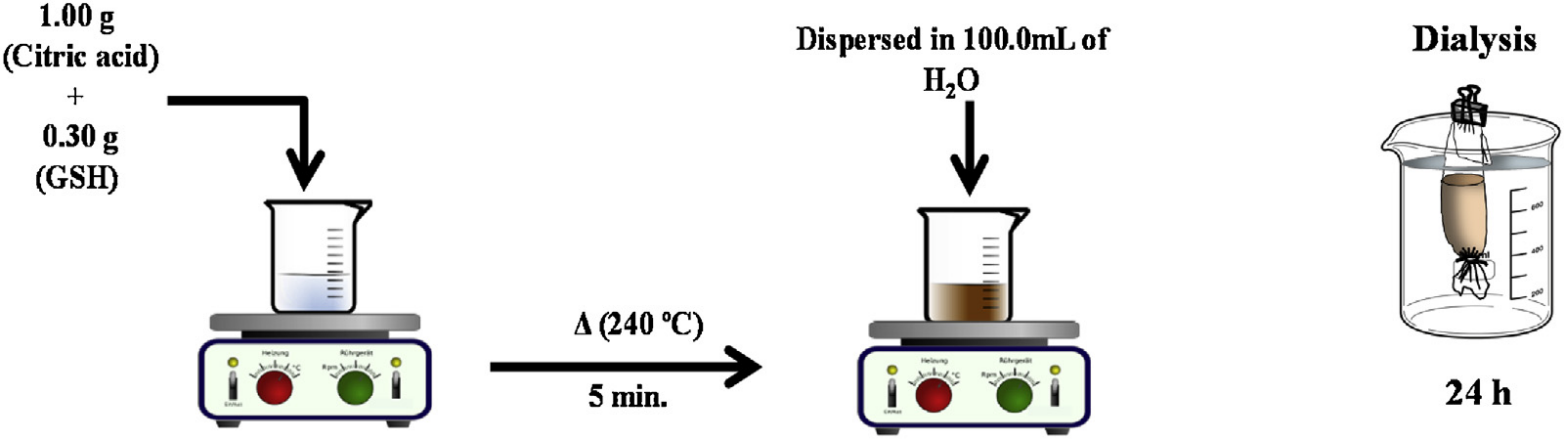
Synthesis procedure aqueous dispersion of GQDs-amino.

The general procedure for the preparation of GQDs by hydro-exfoliation has previously been reported [3]. Amino-GQDs were synthesized by heating a mixture of 1.0 g citric acid and 0.3 g glutathione at 240 °C until a brownish molten mixture was obtained (within 2–5 min). This melt was added to 100 mL of ultrapure water at room-temperature. After passing it through a 0.22 μm syringe filter, a clear pale yellow aqueous dispersion was obtained, which was further dialyzed for 24 h using a dialysis membrane (retained molecular weight of 3.5 kDa) to obtain an aqueous dispersion of amino-GQDs. See Fig. 1 for an illustration of the procedure. Based on total carbon measurements on a Carbon Analyzer model TOC-VCPN (Shimadzu, Japan), the total carbon present in this synthesis dispersion was 120 mg L^−1^.

### Synthesis of a AuNP-amino-GQD-CTAB platform

**Fig. 2:**
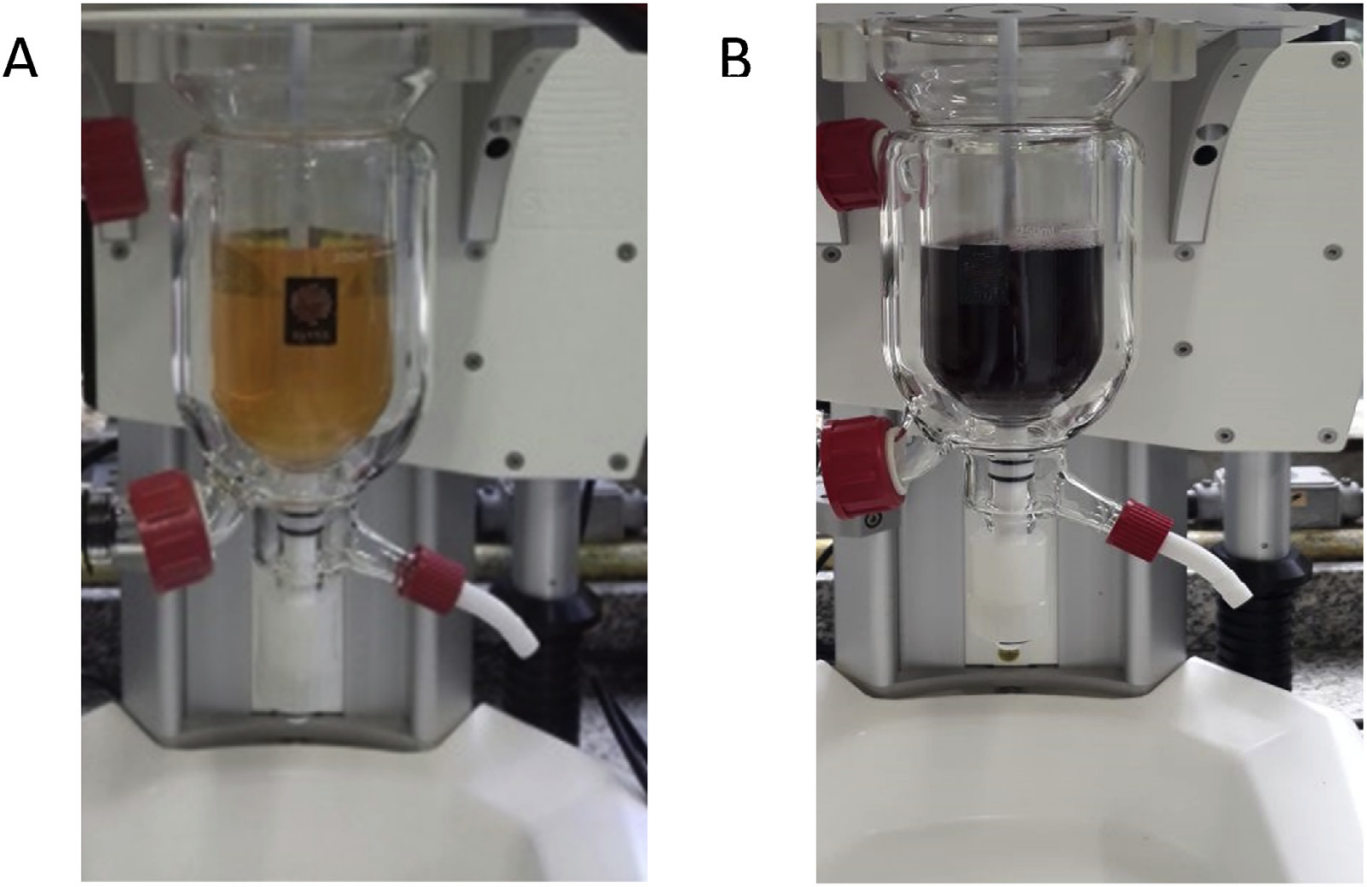
Image of the gold nanoparticle synthesis (AuNP-GQDs-amino) made in the reactor. (A) Mixing the reactants before of addition of NaBH_4_. (B) AuNPs after addition of NaBH_4_.

The AuNP-amino-GQD-CTAB dispersion was prepared adding 3.25 mL of a 23 mmol L^−1^ aqueous solution of hydrogen tetrachloroaurate(III) hydrate into a glass reaction vessel containing 125 mL of water and 125 mL of a mmol L^−1^ aqueous CTAB solution and 0.75 mL of the synthesized amino-GQD dispersion. This mixture was vigorously and continuously stirred (5 min) using a Teflon rod before adding 830 μL of freshly prepared aqueous NaBH_4_ (0.4 mol L^−1^) solution to promote the reduction of [AuCl_4_]– to form AuNPs (See Fig. 2).

### Preparation of kanamycin-imprinted polymer

**Fig. 3:**
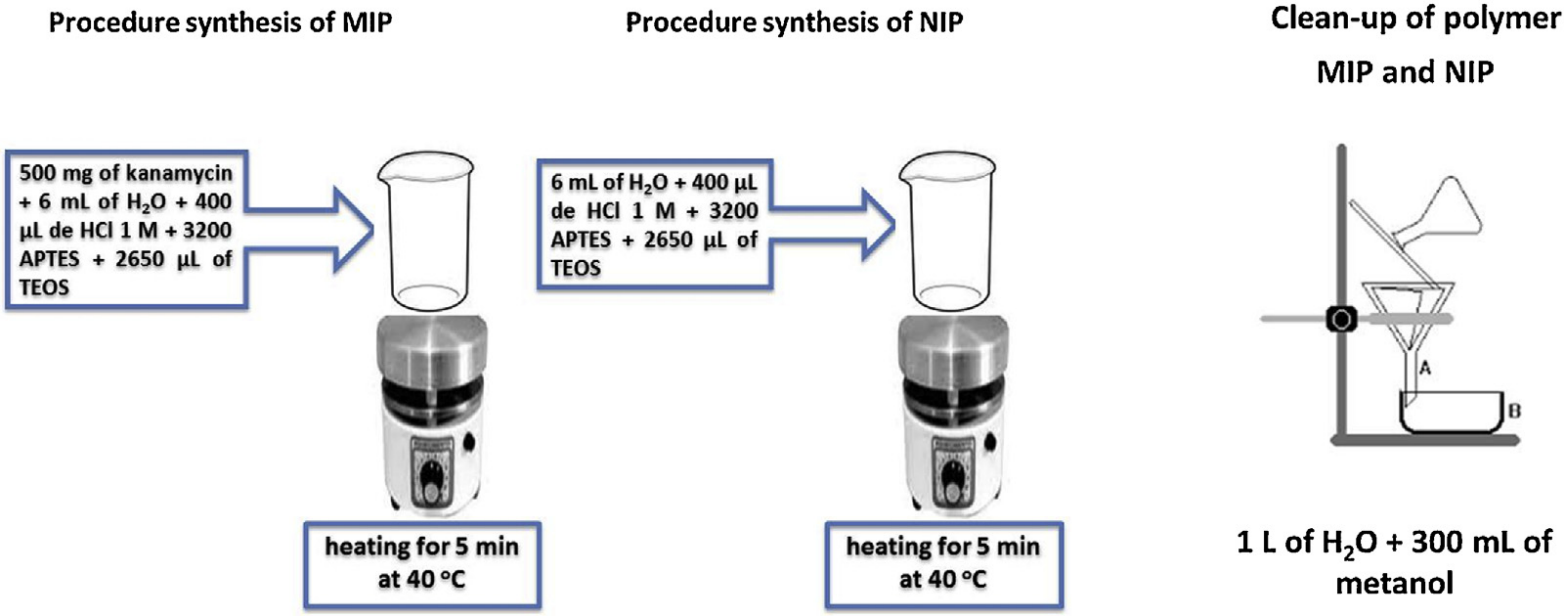
Steps for the synthesis and cleaning of the molecularly imprinted polymer (MIP) and non-printed polymer (NIP).

**Fig. 4:**
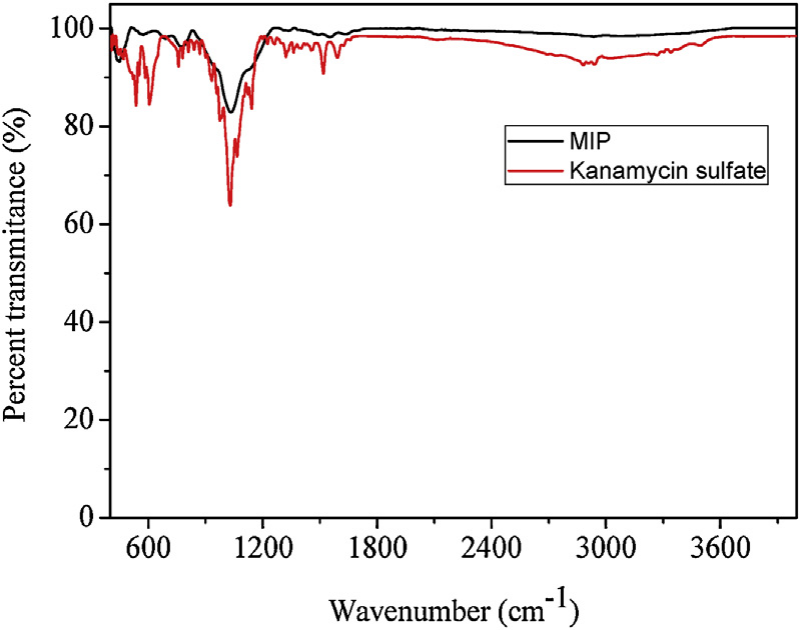
Infrared spectrum of kanamycin sulfate (red) and MIP after clean-up to remove the kanamycin template (black).

**Fig. 5:**
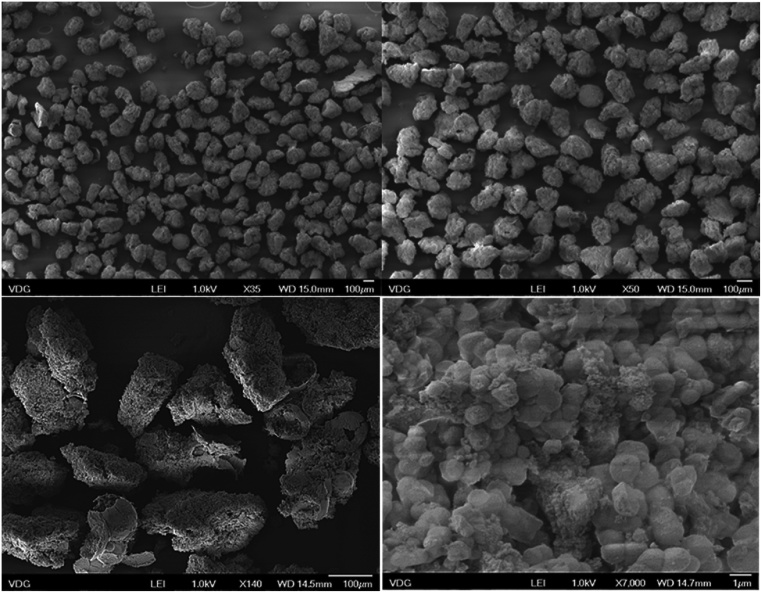
Scanning electron microscopy of the sol-gel matrix kanamycin-MIP (fraction collected in the sieve of 150 μm) using different amplifications: (A) 35 times, (B) 50 times, C) 140 times and D) 7000 times.

A kanamycin-imprinted polymer (using kanamycin sulfate as a template) was synthesized using a sol-gel process [4]. Initially, 500 mg kanamycin sulfate was dissolved in 6 mL deionized water, before 400 μL of 1 mol L^−1^ aqueous HCl solution (as a catalyst), 3200 μL of APTMS and 2650 μL TEOS were added. The mixture was heated to 40 °C until it has just turned turbid. The temperature was then kept at 40 °C with controlled stirring for 5 min. The obtained gel was cooled to room temperature and maintained at room-temperature for 12 h to ensure dryness of the material. After this period, the kanamycin-MIP was crushed using a mortar and pestle, before it was washed with 1 L warm deionised water, followed by 300 mL of methanol to remove kanamycin sulfate. This cleaning process was repeated two more times to ensure complete removal of the template from the polymer. The total extraction of kanamycin sulfate of the MIP was monitored through the photoluminescent response of the AuNP-Amino-GQD-CTAB nanoprobe. The clean polymer was left to dry in a desiccator and then passed through a molecular sieve to obtain regular particles with diameters from 106 to 150 μm. The MIP was stored at room temperature. The corresponding non-imprinted polymer (NIP) was prepared in the same manner but without adding the template. An illustration showing the steps of polymer synthesis and its cleaning process is in Fig. 3. The infrared spectrum of the MIP (after cleaning) and the one of the template (kanamycin sulfate) are shown in Fig. 4. The infrared spectrum of the MIP does not present the characteristic peaks of kanamycin after it was submitted to cleaning, but the characteristic infrared bands of the polymer are dominant: 3307 cm^−1^ (NH_2_ from the APTMS); 2956–2889 cm^−1^ (CH_2_ from the APTMS); 1120 and 1033 cm^−1^ (stretching of C—O from ether and Si—O—Si stretching) and 781 cm^−1^ (Si—C stretching). The micrographs of the produced MIP (after cleaning) are shown in Fig. 5. The micrographs corroborated the diameter of the fraction of MIP chosen to perform extraction.

### Preparation of working dispersions of AuNP-amino-GQDs-amino-CTAB

Working dispersions of AuNP-amino-GQDs-CTAB analytical probe were prepared by placing 1.50 mL of the dispersion in a 5.00 mL volumetric flask. Then, a volume (μL order) of a standard or a sample of kanamycin was added before adjusting the final volume to 5.00 mL with ultrapure water. Working dispersions containing kanamycin sulfate were allowed to stand at room temperature for 30 min prior to measurement. In preparing the blank dispersions, the final volume was adjusted with water only.

### Preparation of kanamycin sulfate standard solutions and sample solutions

Stock standard solutions of analyte were prepared at 1.0 × 10^−2^ mol L^−1^ and at 1.0 mmol L^−1^ by the direct dilution of appropriate amounts of kanamycin sulfate in ultrapure water. Less concentrated standard solutions were prepared by diluting the stock solutions with water. Sample solutions of the pharmaceutical formulations and samples of yellow fever vaccine were prepared only by successive dilutions of the original sample, prior to SPE.

### SPE using MIP

**Fig. 6:**
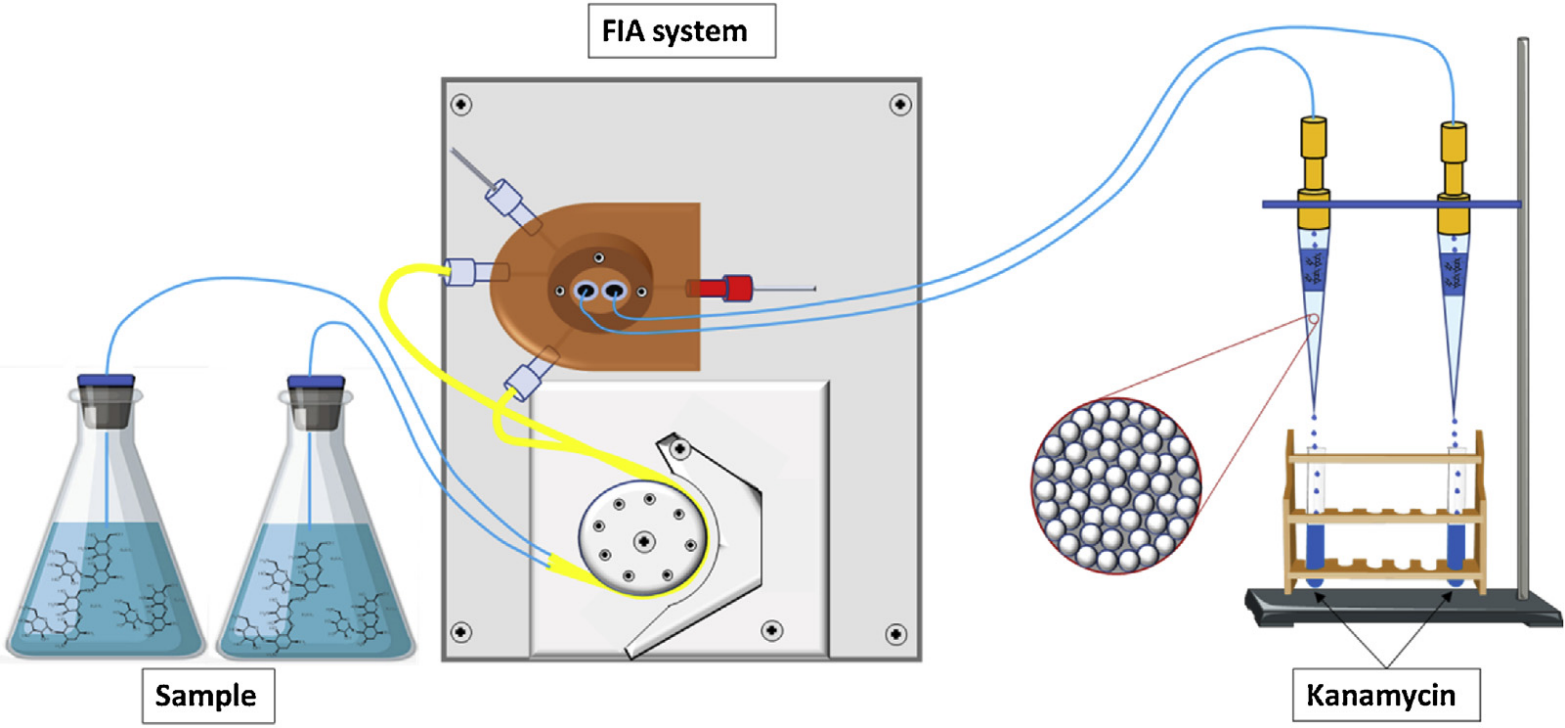
Semi-automated solid-phase extraction.

The cartridges used for SPE were prepared using 70 mg of the kanamycin-MIP which were packed inside a 1 mL plastic micropipette tip containing a little piece of wool near the tip to work as a membrane, allowing the solution to percolate but keeping the MIP particles packing inside the tip [4]. This whole SPE process was semi-automatized with the help of a flow injection system (Fig. 6).

A 40-μL aliquot of sample was loaded into the SPE cartridge, previously conditioned with water. An equilibration time of 30 min was established to let the analyte interact with the polymer before passing 5 mL of ultrapure water through the cartridge at 1 mL min-1 using the flow injection system. This step was to remove any drug or vaccine excipients. The sample was finally eluted using 1 mL of HCl solution (pH 3.5). The total volume obtained from the elution was adjusted to pH 4.5 by adding NaOH 0.01 mol L^−1^ before an aliquot was mixed with the working dispersion to achieve a final probe volume of 5.00 mL with ultrapure water. After each extraction step with the samples, the cartridge was subjected to a clean-up step using 5 mL of warm water and 5 mL of methanol before it was used in subsequent extraction. A single kanamycin-MIP SPE cartridge can be used several times.

### Luminescence measurements

The working dispersion was transferred to 1 cm optical path quartz cuvettes to perform photoluminescence measurement at 345/425 nm. When emission spectra were made, the scan rate was 1000 nm min^−1^. Signals obtained by the interaction of kanamycin sulfate with the probe (L) were normalized by their respective blank signals (L_0_) in order to establish an increasing ratio between the normalized signal of (L−L_0_)/L_0_) and kanamycin sulfate concentration. Thus, the linear range for quantification was modeled by the general equation below, where *m* is the sensitivity of the analytical plot constructed using standards mixed with the working dispersion.

(L−L_0_)/L_0_) = *m* [kanamycin]

**Fig. 7:**
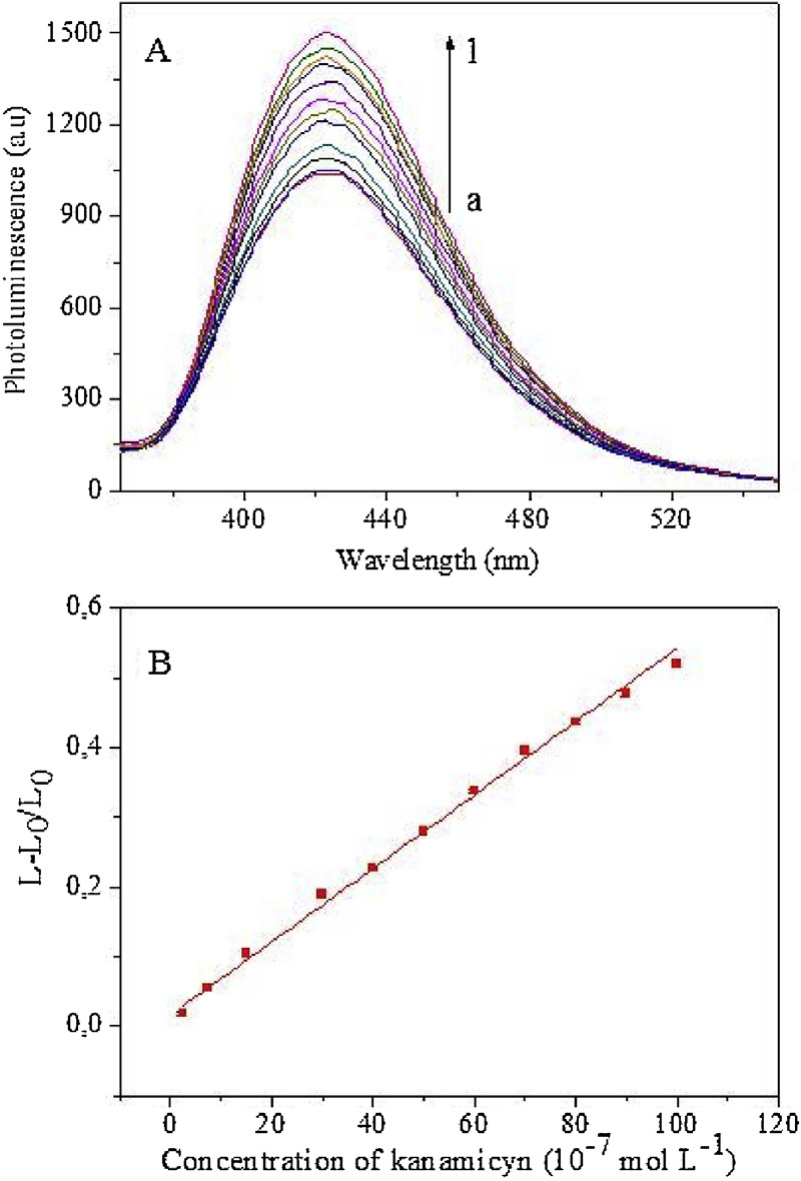
(A) Photoluminescence from the AuNP-amino-GQD-CTAB probe with increasing concentrations of kanamycin: (a) 0 (b) 0.25, (c) 0.75, (d) 1.5, (e) 3.0, (f) 4.0, (g) 5.0, (h) 6.0, (i) 7.0, (j) 8.0, (k) 9.0, (l) 10 μmol L^−1^; (B) Analytical calibration plot based on the photoluminescence enhancement of AuNP-amino-GQD-CTAB.

Fig. 7(A) shows the photoluminescence signal obtained at the AuNP-amino-GQD-CTAB probe in the presence of 0.25–10 μmol L^−1^ kanamycin sulfate. Fig. 7(B) shows a linear (L−L_0_)/L_0_ versus kanamycin sulfate concentration analytical curve. A limit of detection of 0.06 μmol L^−1^ and a limit of quantification of 0.20 μmol L^−1^ kanamycin sulfate were estimated. In terms of mass (considering the sample volume added to the probe), the method can quantify down to 1.4 μg of kanamycin sulfate. Using the analytical curve, the kanamycin sulfate concentration in sample I (fortification of 20 μmol L^−1^ equivalent to 12 μg mL^−1^) was properly recovered (102%). The sample fortified was submitted to the SPE procedure and the result was corrected by a blank study (non-fortified sample).
